# A versatile platform technology for recombinant vaccines using non-propagative human parainfluenza virus type 2 vector

**DOI:** 10.1038/s41598-019-49579-y

**Published:** 2019-09-09

**Authors:** Junpei Ohtsuka, Masayuki Fukumura, Wakako Furuyama, Shujie Wang, Kenichiro Hara, Mitsuyo Maeda, Masato Tsurudome, Hiroko Miyamoto, Aika Kaito, Nobuyuki Tsuda, Yosky Kataoka, Akira Mizoguchi, Ayato Takada, Tetsuya Nosaka

**Affiliations:** 1 Department of Microbiology and Molecular Genetics, Mie University Graduate School of Medicine, Tsu, Japan; 2 Research Center for Development of Recombinant VLP Vaccines, Research Institutes of Excellence, Mie University, Tsu, Japan; 3 BioComo Inc., Komono, Mie, Japan; 4 Division of Global Epidemiology, Research Center for Zoonosis Control, Hokkaido University, Sapporo, Japan; 5 Department of Neural Regeneration and Cell Communication, Mie University Graduate School of Medicine, Tsu, Japan; 6 Multi-Modal Microstructure Analysis Unit, RIKEN-JEOL Collaboration Center, Kobe, Japan; 7 Present Address: Laboratory of Virology, National Institute of Allergy and Infectious Diseases, National Institutes of Health, Rocky Mountain Laboratories, Hamilton, MT USA; 8 Present Address: Project Division of ALA Advanced Medical Research, The Institute of Medical Science, The University of Tokyo, Tokyo, Japan; 9 Present Address: Department of Biomedical Sciences, College of Life and Health Sciences, Chubu University, Kasugai, Japan; 10 Present Address: Department of Physiology, Mie University Graduate School of Medicine, Tsu, Japan

**Keywords:** Vaccines, Viral vectors

## Abstract

Ectopic protein with proper steric structure was efficiently loaded onto the envelope of the *F* gene-defective BC-PIV vector derived from human parainfluenza virus type 2 (hPIV2) by a reverse genetics method of recombinant virus production. Further, ectopic antigenic peptide was successfully loaded either outside, inside, or at both sides of the envelope of the vector. The BC-PIV vector harboring the Ebola virus *GP* gene was able to elicit neutralizing antibodies in mice. In addition, BC-PIV with antigenic epitopes of both melanoma *gp100* and *WT1* tumor antigen induced a CD8+ T-cell-mediated response in tumor-transplanted syngeneic mice. Considering the low pathogenicity and recurrent infections of parental hPIV2, BC-PIV can be used as a versatile vector with high safety for recombinant vaccine development, addressing unmet medical needs.

## Introduction

Life-threatening viruses such as Ebola virus (EBOV), SARS coronavirus, and avian influenza virus, emerge unexpectedly. A platform for the rapid generation of recombinant vaccines is required for mankind. Human parainfluenza virus type 2 (hPIV2) is a negative-stranded paramyxovirus with little pathogenicity for healthy adults. A reverse genetics method to generate clonal populations of recombinant RNA viruses enabled us to use hPIV2 as a vector for efficient transgene expression^1–3^. Since hPIV2 induces no alterations of the host genome and causes repeated infection throughout a human’s lifetime due to incomplete immunity against hPIV2^4^, it is an ideal vector for delivering a vaccine antigen.

**Figure 1:**
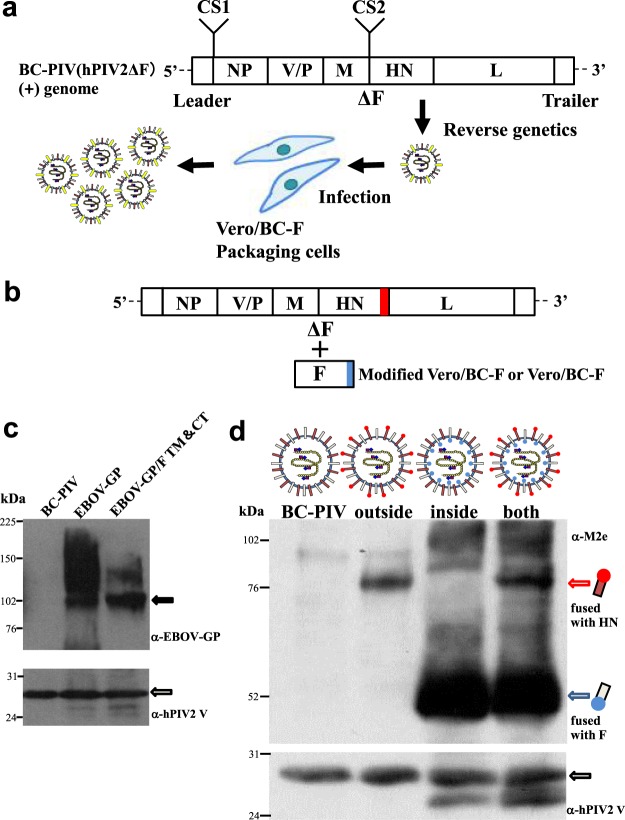
Platform technology for ectopic antigen expression using BC-PIV. (**a**) Graphic depiction of the production of recombinant vaccines. Antigen genes are inserted into the cloning site (CS) 1 and/or CS2 of BC-PIV, and the resultant vectors are produced by the reverse genetics method^1–3,5^. (**b**) Another method for antigen expression as a fusion protein with the C-terminus of HN of BC-PIV, and/or that of F from packaging cells. (**c**) A Western blot analysis of EBOV-GP on BC-PIV using 1 × 10^6^ particles/lane. The authentic or hybrid EBOV *GP* gene was inserted into CS2 of BC-PIV. F TM&CT, transmembrane and cytoplasmic tail regions of hPIV2 F. (**d**) A Western blot analysis of M2e peptide fused with hPIV2 HN and/or F on BC-PIV using 1 × 10^6^ particles/lane.

We previously generated a non-propagative hPIV2 vector by deleting the *F* gene, which is essential for viral-cell membrane fusion and subsequent viral proliferation (hPIV2ΔF^3^; BC-PIV) and the stable packaging cell line Vero/BC-F^5^ (Fig. 1a). BC-PIV is able to induce gene expression nearly 100 times more efficiently than a conventional retroviral vector in human dendritic cells^3^.

We herein report that not only does BC-PIV vector highly express ectopic antigen in infected cells^3,5^, but it also displays a large amount of ectopic antigen on the viral envelope, enabling it to skip the translation process in host cells, similar to virus-like particles (VLPs). The incorporation of foreign proteins into virus particles was previously reported^6^. However, our platform technology confers controlled localization of the exogenous product across the viral envelope and proper steric structure of the exogenous protein expressed on the envelope to induce neutralization antibodies against it, by using the replication-defective and highly efficient vector, BC-PIV.

## Results

### Generation of BC-PIV/EBOV-GP

**Figure 2:**
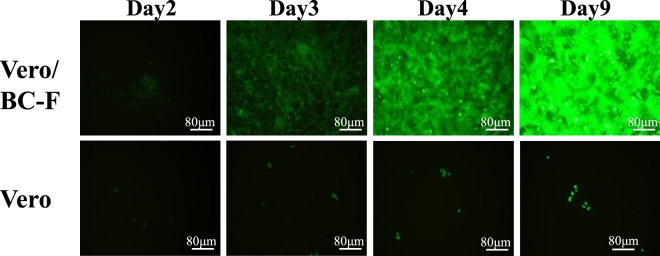
BC-PIV/EGFP-EBOV-GP proliferates only in the presence of hPIV2 F protein. The *EGFP* gene was inserted into the CS1 of BC-PIV/EBOV-GP and designated BC-PIV/EGFP-EBOV-GP. Vero/BC-F cells^5^, a packaging cell line stably expressing hPIV2 F protein, were infected with BC-PIV/EGFP-EBOV-GP at an MOI of 0.3. Vero cells were also infected with the same virus at an MOI of 0.3. BC-PIV/EGFP-EBOV-GP does not proliferate in Vero cells; however, it does proliferate in Vero/BC-F cells, resulting in a titer of 1 × 10^8^ TCID_50_/mL. The EGFP expression was visualized by fluorescence microscopy (CKX41; OLYMPUS, Tokyo, Japan).

**Figure 3:**
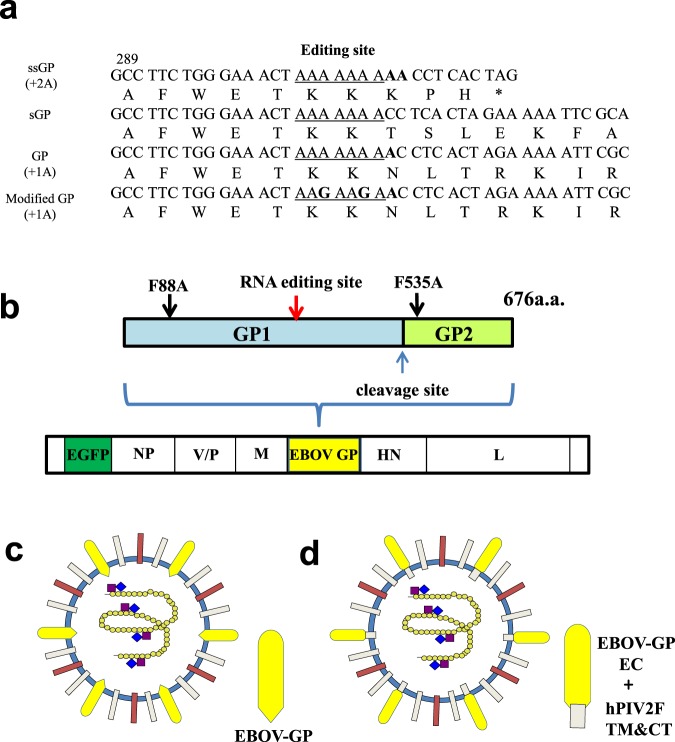
Generation of BC-PIV/EGFP-EBOV-GP. (**a**) Mutagenesis at the RNA editing site^11^ results in the production of only the full-length form of the GP protein, composed of 676 amino acids. (**b)** A schematic illustration of BC-PIV/EGFP-EBOV-GP. Double mutations (F88A and F535A) abolish the GP-mediated viral growth. (**c**) A schematic illustration of the vector with the full-length EBOV-GP. (**d**) A schematic illustration of the vector with a hybrid protein composed of the extracellular (EC) domain of EBOV-GP with transmembrane (TM) and cytoplasmic tail (CT) regions of hPIV2 F.

First, we generated EBOV vaccine using the full-length EBOV *GP* gene. It should be noted that, depending on the property of the ectopically expressed envelope protein, the ability to proliferate in the non-propagative recombinant vector can be regained in the absence of the cognate envelope protein. This is what happens with EBOV-GP^7,8^. However, double mutations^9,10^ in the *GP* gene (F88A/F535A) completely abrogated the GP-mediated proliferation of the recombinant BC-PIV *in vitro* (Figs 2 and 3b) while retaining the antigenicity. In addition, mutations within the *GP1* region were introduced to prevent RNA editing^11^ without affecting the amino acid sequence (Fig. 3a,b). The resultant vaccine BC-PIV/EBOV-GP incorporated a large amount of GP protein on the viral particles, as shown in the Western blot analysis (Fig. 1c).

Consistent with previous reports^6–8,12^, transmembrane and cytoplasmic regions of the envelope protein of the cognate virus are not necessarily required for the efficient incorporation of exogenous protein onto the virions compared with that of an authentic form of the exogenous protein (Figs 1c and 3c,d). The band at around 100 kDa is weaker in the authentic EBOV-GP than in the hybrid form (extracellular domain: EBOV-GP, transmembrane domain and cytoplasmic tail: hPIV2 F), while the upper smear bands are much stronger in the authentic form than in the hybrid form. These differences may be derived from different levels of glycosylation of incorporated EBOV GP^13^.

### Visualization of the ectopically expressed antigen on the vector particle

**Figure 4:**
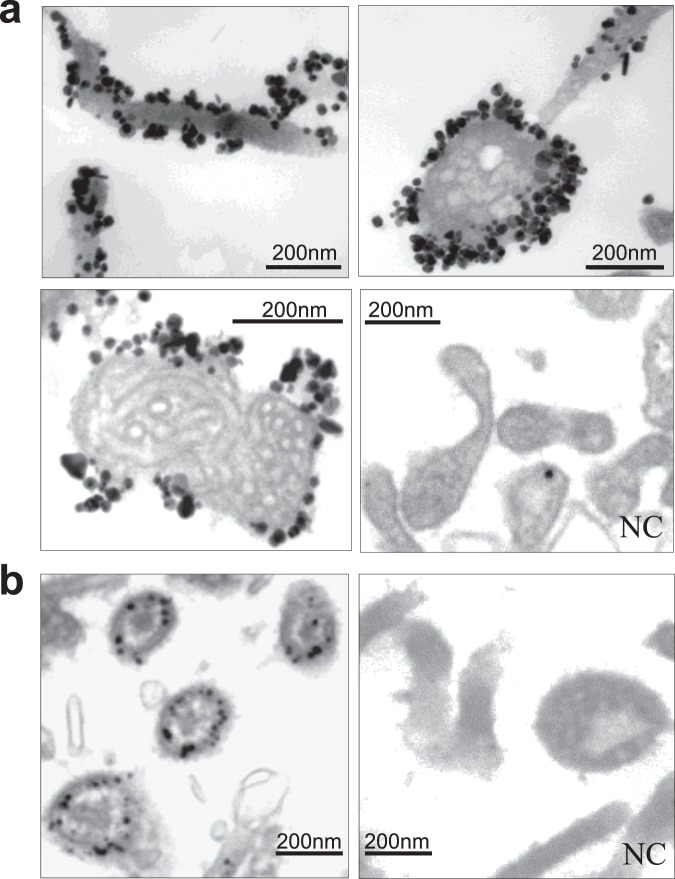
Immunoelectron microscopy for antigens on vector particles. (**a**) BC-PIV/EBOV-GP. (**b**) BC-PIV/M2e (inside type). NC, mouse IgG was used as a negative control.

Immunoelectron microscopy using an anti-EBOV-GP neutralizing monoclonal antibody recognizing a conformational epitope^14^ in its trimeric, pre-fusion form showed that the native form of the GP protein is abundantly present on the viral surface (Fig. 4a). Each colloidal gold corresponds to each molecule (GP1-GP2 heterodimer) of the GP protein. Coiled ribonucleocapsid complex of hPIV2 is clearly visible inside the virion.

### Induction of neutralizing antibody against EBOV

**Figure 5:**
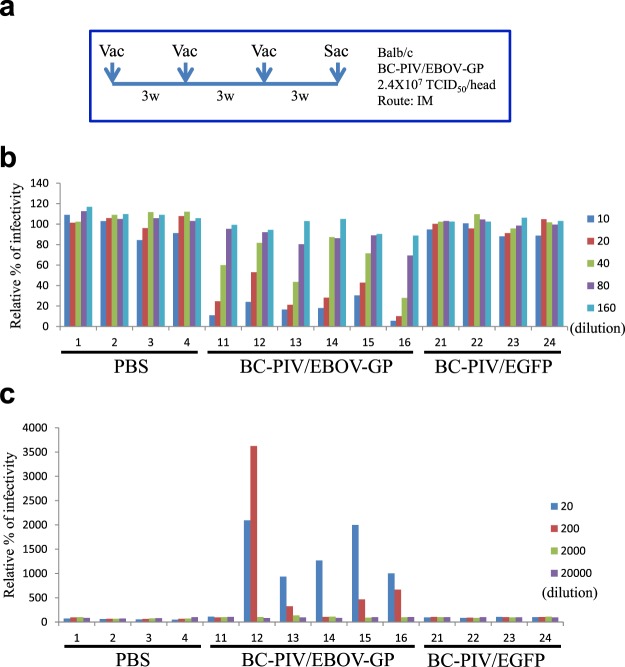
Induction of neutralizing antibody in mice vaccinated with BC-PIV/EBOV-GP. (**a**) Protocol for vaccination of mice. Vac, vaccination; Sac, sacrifice; IM, intramuscular injection. (**b**) Neutralization tests against EBOV-GP-pseudotyped virus using vaccinated mouse sera. (**c**) ADE tests of pseudotyped EBOV infection using the mouse sera used in **b**. All data are representative of three independent experiments. Each number represents the ID number of the mice.

The ability of the EBOV vaccine to induce efficient humoral immunity (IgG_1_ and IgG_2a_) in mice was revealed by an enzyme-linked immunosorbent assay measuring the specific antibody titer of the sera (>100,000 × dilution, data not shown) and a pseudotype virus-based inhibition assay^15^ measuring the neutralizing antibody titer of the sera (Fig. 5a,b). These titers were comparable to those induced by Ebola VLPs in mice^16^, or by chimeric hPIV3 bearing the EBOV GP in guinea pigs^8^. Antibodies elicited by BC-PIV/EBOV-GP also induced antibody-dependent enhancement (ADE)^17^ of pseudotyped EBOV infection (Fig. 5c). The ADE activities induced by BC-PIV/EBOV GP were also comparable to those induced by Ebola VLPs in mice^16^ and those of the sera from monkeys that survived challenge with EBOV^16^. A sufficient neutralizing activity of the sera indicates that the ADE effects were overcome by the inhibitory effects of the sera in virus infection. Replication-competent vesicular stomatitis virus (VSV)/EBOV-GP was previously reported to protect against lethal EBOV challenge in nonhuman primates^7,18^, thereby CD4+ T cell-depleted animals succumbed to EBOV infection due to a lack of induction of specific antibodies while the CD8+ T cell-depleted ones survived^19^. These findings suggested that antibodies play an important role in VSV/EBOV-GP-mediated protection against EBOV challenge. Although BC-PIV/EBOV-GP would not propagate *in vivo* in contrast to VSV/EBOV-GP, toxicity and protection studies in primates will still be required in the future.

### Generation of BC-PIVs carrying antigenic peptides

We also generated vaccines against antigenic peptide via another strategy (Fig. 1b). We made three types of recombinant vaccines against the ectodomain of matrix protein 2 (M2e) peptide of influenza A virus, designated as BC-PIV/M2e. In these vaccines, the M2e peptide 2–25 (SLLTEVETPIRNEWGCRCNDSSDP) was loaded either inside, outside, or at both sides of the viral envelope of BC-PIV by fusing the *M2e* peptide gene with the C-terminus of hPIV2 *F* gene or with that of the *HN* gene. For the construction of the inside version, the *M2e* peptide gene was fused with the *F* gene and stably transfected into Vero cells as packaging cells to produce the recombinant vectors. For the outside version, the *M2e* peptide gene was fused with the *HN* gene in the hPIV2 genome and used to produce the recombinant vectors in the Vero/BC-F cells or genetically modified Vero cells expressing M2e-fused F protein. Since F and HN proteins are oppositely oriented across the viral membrane, these strategies work (Fig. 1b,d). The M2e peptide was detected by a Western blot analysis in each case (Fig. 1d). The bands of the inside peptide are more abundant than those of the outside peptide. This may have resulted from differences in the expression of these peptides: the inside peptide was derived from its abundant and stable expression in the packaging cells, while the outside peptide was derived from transcripts from the vector. However, the possibility of better incorporation or enhanced stability of the inside-peptide, compared with the outside peptide, cannot be excluded.

The inside-type M2e peptide-fused protein was shown to be localized within the viral particle by immunoelectron microscopy (Fig. 4b). The inside type would be suitable for inducing cellular immunity after cross-presentation, escaping neutralization by antibodies.

### Induction of CD8+ lymphocytes *via* BC-PIV carrying antigenic peptides against melanoma in a syngeneic mouse tumor model

**Figure 6:**
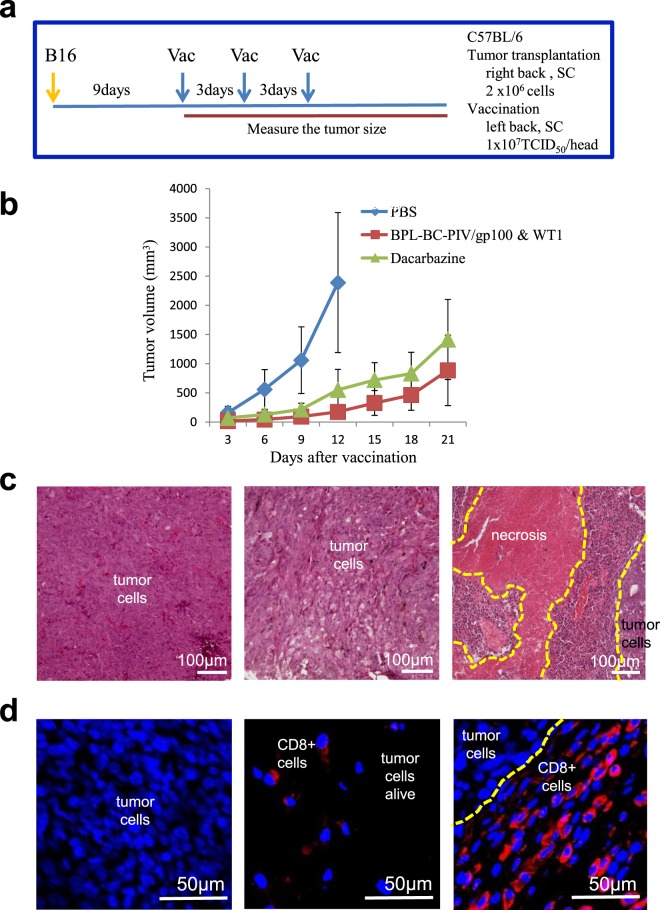
The accumulation of CD8+ lymphocytes and decreased tumor growth in melanoma-transplanted mice after vaccination with bivalent BC-PIV/gp100 & WT1. (**a)** The protocol for transplantation of mouse melanoma B16 cells and subsequent vaccination of syngeneic mice. Vac, vaccination; SC, subcutaneous injection. (**b)** Effects of the vaccination. BC-PIV/gp100 & WT1 harbors both a melanoma gp100-specific CTL epitope KVPRNQDWL fused with hPIV2 HN (the peptide is located outside the viral membrane, see Fig. 1b,d) and a WT1-specific CTL epitope RMFPNAPYL fused with hPIV2 F (the peptide is located inside the viral membrane). The anticancer drug dacarbazine was intraperitoneally injected (100 mg/kg) at the same timing as each vaccination in order to compare the effects. BC-PIV/gp100 & WT1 was treated with 0.01% β-propiolactone (BPL) to inactivate the viral genome before injection^3^. The 0.01% BPL treatment was confirmed to be sufficient to abolish the expression of the transgene in BC-PIV (data not shown). Five mice per group were used, and the means ± SD are shown. PBS-injected mice were euthanized at day 12. (**c**) Hematoxylin and Eosin staining of the transplanted tumors. Left, PBS-injected group at day 12; middle, dacarbazine-administered group at day 21. Tumor cells were diffusely dying; right, vaccinated group with BC-PIV/gp100 & WT1 at day 21. The center was necrotic, and massive infiltration of lymphocytes was found at the tumor boundary. (**d**) The accumulation of CD8+ T-lymphocytes towards the tumor cells in the mice vaccinated with BC-PIV/gp100 & WT1. CD8 was stained with Cy3. Blue regions correspond to nuclei. IDs are the same as in c. CD8+ cells were not found in the PBS-injected group, and a few CD8+ cells were scattered in the dacarbazine-administered group. However, CD8+ cells accumulated at the tumor boundary in the vaccinated group.

Cytotoxic T lymphocyte (CTL) induction is another prerequisite as a vaccine vector. We made anti-cancer vaccines expressing CTL epitopes of melanoma gp100 and WT1 using the strategy shown in Fig. 1b, and tested the effects of these vaccines after inactivation with β-propiolactone^3^. Vaccination of the mice with gp100 (outside) and WT1 (inside) peptides loaded on the BC-PIV envelope suppressed melanoma growth with CD8+ tumor-infiltrating lymphocytes (Fig. 6).

## Discussion

It is noteworthy that mice are not permissive for hPIV2 transcription and replication^20^. The results showing the efficacy of the vaccine in mice in the present study suggest the applicative potential of hPIV2 vector in humans that allow much more efficient transcription of BC-PIV, in addition to its use as inactivated vaccine without transcription.

The platform technology we have developed is based on the non-propagative hPIV2, which has ideal properties for the efficient delivery of ectopically expressed vaccine antigens. To achieve high yields of the non-propagative vector, it is essential to establish a packaging cell line stably producing the deleted gene product. Using Vero/BC-F packaging cells, we were able to obtain a maximum titer of 6 × 10^8^ TCID_50_ (median tissue culture infectious dose)/mL of BC-PIV/EGFP^5^. In contrast to virosome technology, the system in the present study does not require complex processes of antigen incorporation into the vector. Our system utilizes self-assembly of viral components such as VLP by reverse genetics, which allows us to create a stable steric structure of the antigen. In addition, BC-PIV was shown to have an intrinsic adjuvant activity^3^ and is capable of delivering the gene and protein through intranasal, transtracheal, subcutaneous, and intramuscular administrations.

Chimeric hPIV3 bearing the EBOV GP, named HPIV3/ΔF-HN/EboGP, was previously reported^8^. However, this virus is replication-competent, as in VSV/EBOV-GP^7^, and the stable packaging cell lines expressing deleted genes have not been established. Similarly, attenuated hPIV1 expressing EBOV GP^12^, a chimeric bovine/human PIV3 vector expressing respiratory syncytial virus F protein^21^, and PIV5-vectored vaccines against human and animal infectious diseases^22^ were also reported. All of these PIV vectors are replication-competent. Although replication-competent recombinant vaccines are beneficial for the massive induction of an antigen unless adverse events occur, replication-defective vaccines are safer. Balancing the efficacy and likelihood of side effects with a vaccine is important.

Our platform technology may be useful for fast-track vaccine development in addressing various emerging infectious diseases and cancers.

## Methods

### Recovery of recombinant BC-PIV vector

Vero/BC-F cells^5^ were transfected with a recombinant BC-PIV (10 μg), together with each expression plasmid encoding the hPIV2 *NP* (2 μg), *P* (0.9 μg), *L* (2 μg), and *T7* RNA polymerase (3 μg) using X-treme GENE HP (Roche, Indianapolis, IN, USA) according to the manufacturer’s instructions. One week later, the supernatant was centrifuged to remove cells and cell debris and transferred to fresh Vero/BC-F cell culture in minimal essential medium (MEM) (Sigma, St. Louis, MO, USA) with 1% heat-inactivated fetal bovine serum (FBS) (GIBCO/Invitrogen, Grand Island, NY, USA) (maintenance medium) for virus propagation. The sequences and detailed construction procedures of the plasmids are available upon request.

### Detection of the ectopically expressed antigen on the virus

Vero/BC-F cells (1.0 × 10^6^) were infected with the recombinant BC-PIV at a multiplicity of infection (MOI) of 0.1, and incubated with maintenance medium for 7 days. Lysates from purified viral particles were subjected to Western blot analyses with an anti-EBOV-GP rabbit polyclonal antibody (Ab) (Sino Biological Inc, Beijing, China), an anti-M2e mouse monoclonal antibody (mAb) (14C2; Abcam, Cambridge, MA, USA), and an anti-hPIV2 V mAb (53-1)^23^, respectively.

### Immunoelectron microscopy

Immunoelectron microscopy using the silver-enhancement technique was performed as described previously^24^. In brief, vector-producing Vero/BC-F cells cultured in a chamber were fixed with 2% fresh formaldehyde and 2.5% glutaraldehyde in 0.15 M sodium cacodylate buffer/2 mM CaCl_2_ (pH 7.4) at room temperature (RT) for 2 h, treated with 0.1 M phosphate buffer (PB) (pH 7.4) containing 4% Block Ace (DS Pharma Biomedical, Suita, Japan), protease inhibitor cocktail tablets (Roche Diagnostics, Mannheim, Germany), and 0.02% saponin (Nacalai USA, Inc., San Diego, CA, USA), and incubated at RT for 20 min. The samples were then incubated with an anti-EBOV GP (KZ52; Absolute Antibody Ltd, Oxford, UK) or the anti-M2e mAb at 4 °C for 2 h, followed by reaction with an anti-mouse IgG Ab coupled with 1.4 nm gold particles (Nanoprobes, Stony Brook, NY, USA) at RT for a further 2 h. The Abs were diluted in 0.1M PB (pH 7.5) containing 0.02% saponin, 1% Block Ace, and protease inhibitor cocktail. The sample-bound gold particles were silver-enhanced at 18 °C for 12 min using an HQ-silver kit (Nanoprobes). The samples were washed, postfixed, dehydrated, and embedded in epoxy resin. From this sample, 75-nm-thick ultrathin sections were cut, stained with uranyl acetate and lead citrate, and then observed with an electron microscope (JEM-1200EX, and JEM-1230; JEOL, Tokyo, Japan).

### Neutralization tests

Zaire Ebola GP-pseudotyped replication-incompetent VSVs containing the *GFP* gene instead of VSV *G* were generated as described previously^15^. Pseudotyped viruses suspended in growth medium were mixed with an equal volume of heat-inactivated mouse serum diluted in the medium and incubated for 30 min at RT. The mixture was then inoculated to confluent Vero E6 cells grown in 96-well plates (1200–1500 infectious units (IUs)/well). Twenty hours later, the GFP-positive cells were counted. Virus infectivity was quantified by comparing the GFP-positive cell numbers, in which the relative percentage of infectivity in the absence of mouse serum was set as 100%. The IUs of replication-incompetent pseudotyped VSVs were determined using Vero E6 cells as described previously^15^.

### ADE tests

Zaire Ebola GP-pseudotyped replication-incompetent VSVs containing *GFP* gene instead of VSV *G* suspended in growth medium were mixed with an equal volume of heat-inactivated mouse serum diluted in the medium and incubated for 30 min at RT. The mixture was then inoculated to K562 cells grown in 96-well plates (30 IUs/well). Twenty hours later, GFP-positive cells were counted. Virus infectivity was quantified by comparing the GFP-positive cell numbers, in which the relative percentage of infectivity in the absence of mouse serum was set as 100%.

### Vaccination of the mice transplanted with tumor cells and immunohistochemistry

B16 melanoma cells were obtained from JCRB Cell bank (Osaka, Japan) and cultured in MEM with 10% FBS. The BC-PIV/gp100 & WT1 viruses were generated in Vero cells stably expressing the hPIV2 F-WT1 peptide via transduction with BC-PIV harboring the hPIV2 *HN-gp100* peptide gene. The viruses were inactivated with 0.01% BPL (FUJIFILM Wako Pure Chemical Corp., Osaka, Japan) at 4 °C for 17 h, followed by inactivation of BPL at 37 °C for 3 h. B16 cells (2 × 10^6^) were subcutaneously injected into the right side of the back of five-week-old female C57BL/6 mice. Nine days after the transplantation of the B16 cells, BC-PIV/gp100 & WT1 (1 × 10^7^ TCID_50_/100 μL of PBS), dacarbazine (100 mg/kg) (Kyowa Hakko Kirin Co Ltd., Tokyo, Japan), and PBS were subcutaneously injected into the left side of the back of the mice three times at an interval of 3 days. The mice for analysis were anesthetized with diethyl ether and perfused via the vascular system with 0.1M PB (pH 7.4) followed by 4% paraformaldehyde in PB. The transplanted tumor mass was resected, fixed with 4% paraformaldehyde in PB at 4 °C for 24 h, cryoprotected with 20% sucrose in PB for 48 h, and frozen at −80 °C with dry ice powder. Sections (10 μm thick) were prepared using a cryostat (Microm HM 560; Microedge Instruments Inc., White Rock, BC, Canada) and reacted with an anti-mouse CD8 rat mAb (1:200 dilution; Abcam, Cambridge, UK) in 0.3% Triton X-100 in PBS (PBS-T) at 4 °C for 24 h. After washing for 30 min (3 washes of 10 min each) with PBS-T, sections were incubated with a Cy3-conjugated anti-rat IgG Ab (1:200; Jackson ImmunoResearch Laboratories Inc, West Grove, PA, USA) at RT for 2 h and washed with PBS-T for 30 min. Sections were then mounted with solution containing 4′,6-diamidino-2-phenylindole (DAPI) (1 mg/mL, Dojindo Laboratories, Tokyo, Japan) and examined using a confocal laser scanning microscope (Digital Eclipse C1; Nikon, Tokyo, Japan).

### Mice

The animal studies were approved by the Animal Care Committees of Mie University (Approved No. 23–33), and all methods were performed under institutional regulations of animal experiments in accordance with the current national guidelines.

## Supplementary information

Suppl Fig 1
