# Supplementary material for: A versatile platform technology for recombinant vaccines using non-propagative human parainfluenza virus type 2 vector
Source: Sci Rep. 2019 Sep 9;9:12901. doi: 10.1038/s41598-019-49579-y (PMC6733870; doi:10.1038/s41598-019-49579-y)

Supplementary information for:

A versatile platform technology for recombinant vaccines using non-propagative human parainfluenza virus type 2 vector

Junpei Ohtsuka[1,2,3], Masayuki Fukumura[1,2,3], Wakako Furuyama[4,7], Shujie Wang[5], Kenichiro Hara[1,8], Mitsuyo Maeda[6], Masato Tsurudome[1,2,9], Hiroko Miyamoto[4], Aika Kaito[5,10], Nobuyuki Tsuda[1], Yosky Kataoka[6], Akira Mizoguchi[2,5], Ayato Takada[4] & Tetsuya Nosaka[1,2]

[1]Department of Microbiology and Molecular Genetics, Mie University Graduate School of Medicine, Tsu, Japan. [2]Research Center for Development of Recombinant VLP Vaccines, Research Institutes of Excellence, Mie University, Tsu, Japan. [3]BioComo Inc., Komono, Mie, Japan. [4]Division of Global Epidemiology, Research Center for Zoonosis Control, Hokkaido University, Sapporo, Japan. [5]Department of Neural Regeneration and Cell Communication, Mie University Graduate School of Medicine, Tsu, Japan. [6]Multi-Modal Microstructure Analysis Unit, RIKEN-JEOL Collaboration Center, Kobe, Japan.
[7]Present address: Laboratory of Virology, National Institute of Allergy and Infectious Diseases, National Institutes of Health, Rocky Mountain Laboratories, Hamilton, MT, USA.
[8]Present address: Project Division of ALA Advanced Medical Research, The Institute of Medical Science, The University of Tokyo, Tokyo, Japan.
[9]Present address: Department of Biomedical Sciences, College of Life and Health Sciences, Chubu University, Kasugai, Japan.
[10]Present address: Department of Physiology, Mie University Graduate School of Medicine, Tsu, Japan.

Correspondence should be addressed to T.N. (nosaka@doc.medic.mie-u.ac. jp) or M.F. (m-fukumura@biocomo.jp).

Original blots in Fig.1

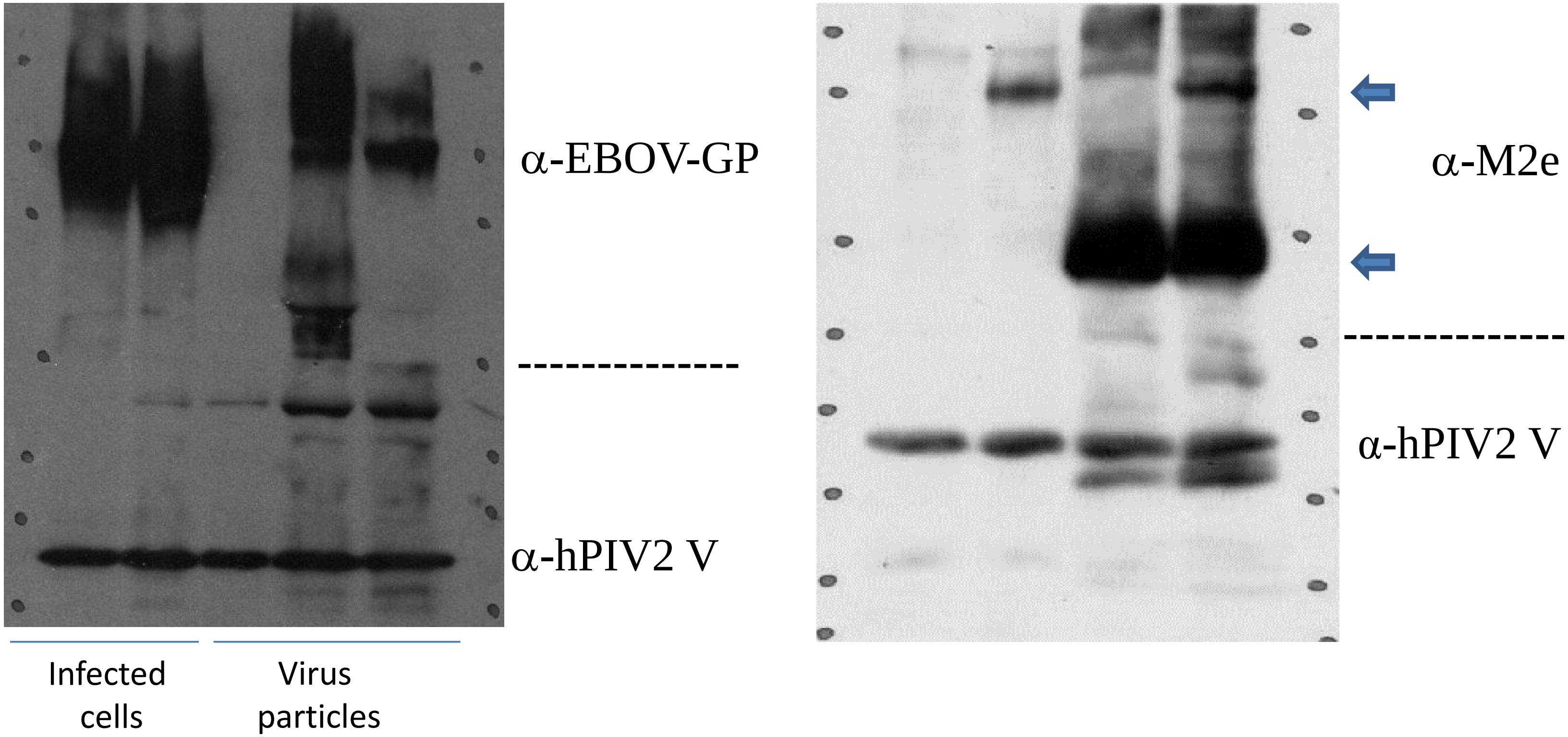

Supplement: Supplementary file 1 — Suppl Fig 1 [file 41598_2019_49579_MOESM1_ESM.pdf]
